# Large Language Model in Automatic Depression Detection

**DOI:** 10.1192/j.eurpsy.2026.11414

**Published:** 2026-07-31

**Authors:** S. H. Ling, W. Chorney

**Affiliations:** 1 School of Medicine, University of Limerick, Limerick; 2 College of Medicine and Health, University College Cork, Cork, Ireland

## Abstract

**Introduction:**

Depression affects over 280 million people globally and is linked to substantial morbidity, mortality, and poor outcomes in comorbid conditions. Despite the availability of validated screening tools, up to 63.6% of cases remain undiagnosed, delaying timely treatment. Automated depression screening using text or voice data has emerged to improve accessibility and detection. Large language models (LLMs) have shown promise in identifying depression from social media, clinical notes, and structured interviews. However, current performance estimates are most likely optimistic due to relying on commercial models.

**Objectives:**

This study evaluates the feasibility and accuracy of using privacy-preserving locally deployed LLMs with realistic size constraints for automated depression detection using clinical dialogue transcripts.

**Methods:**

We employed the Distress Analysis Interview Corpus–Wizard-of-Oz (DAIC-WOZ) dataset containing therapist–patient transcripts labelled for depression. Several open-source LLMs under 15 billion parameters (Gemma3, Qwen3, DeepSeek-R1, Phi3, and LLaMA3.2) were deployed locally via Ollama. Each model received a standardized system prompt to classify each transcript as “depressed” (1) or “not depressed” (0). Predictions were repeated ten times per model. Accuracy, sensitivity, specificity, precision, F1 score, and Cohen’s kappa were calculated with 95% confidence intervals using Scikit-learn and Scipy (α = 0.05).

**Results:**

Each model is trained and validated on the Distress Analysis Interview Corpus/Wizard of Oz dataset using locally deployed LLMs to preserve privacy. Best performing models achieved 67.7% accuracy, 46.2% precision, 72.9% specificity (deepseek-r1:14b), 92.9% sensitivity (gemma3:1b), and 56.4% F1 (gemma3:12b).

**Image 1:**
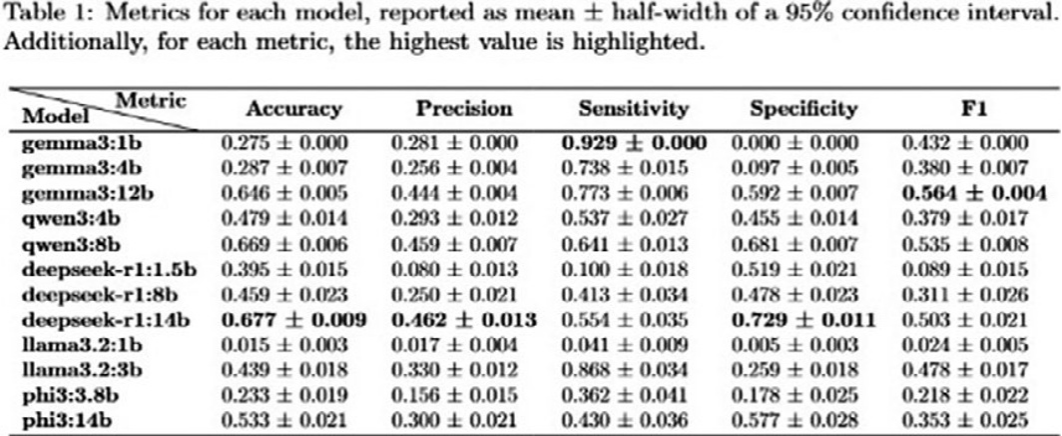
Image 1: Long description.

**Image 2:**
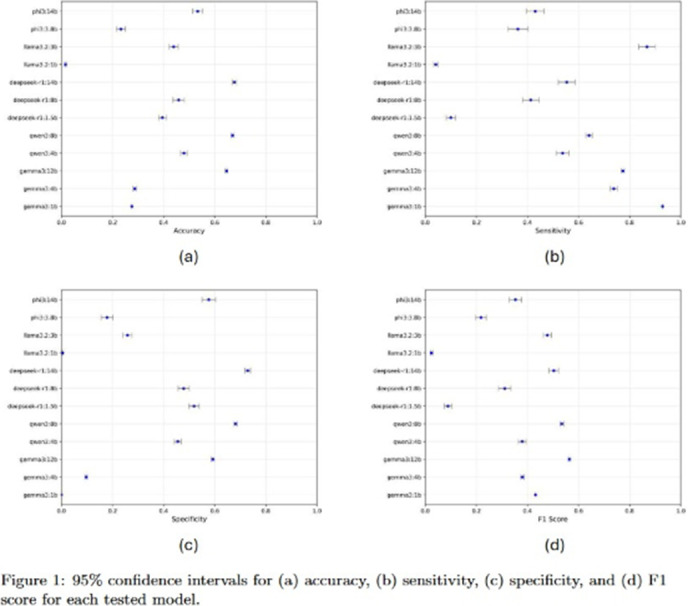
Image 2: Long description.

**Image 3:**
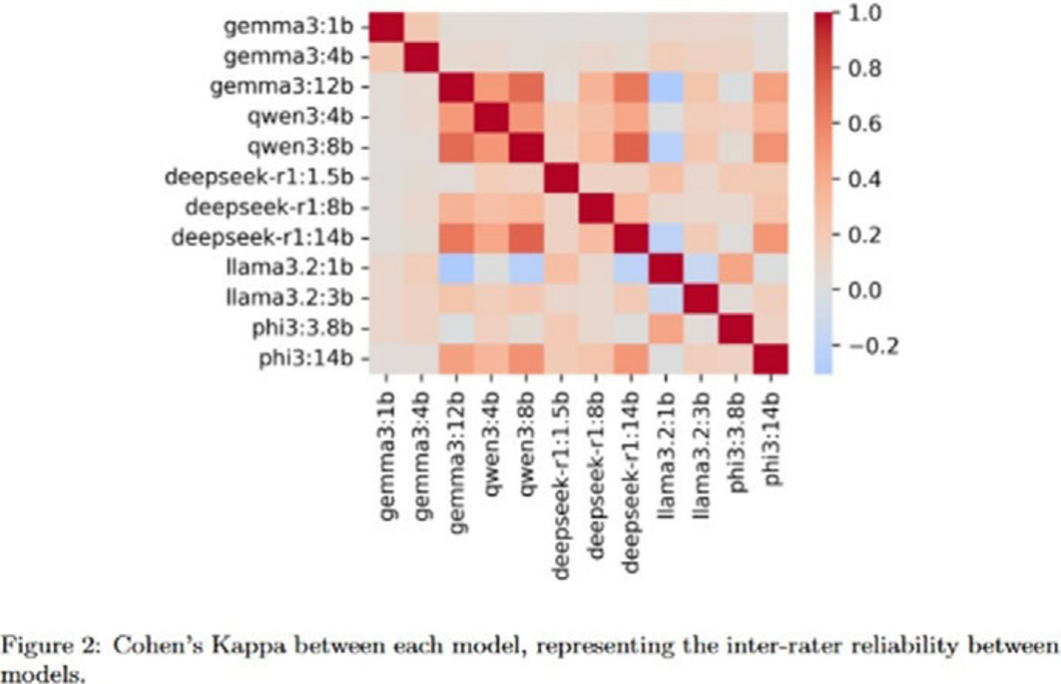
Image 3: Long description.

**Conclusions:**

The results demonstrated that current local LLMs are not adequate for clinical utility in automatic depression screening. Future studies are required to fine tune locally operated LLMs for depression screening.

**Disclosure of Interest:**

None Declared

## Long descriptions

### Image 1: Long description

The table consists of six columns. The first column lists the Model names, and the subsequent columns list metrics: Accuracy, Precision, Sensitivity, Specificity, and F 1. Each cell contains a mean value plus or minus a 95 percent confidence interval half-width.

* gemma 3: 1 b: 0.275 plus or minus 0.000, 0.281 plus or minus 0.000, 0.929 plus or minus 0.000 (highest Sensitivity), 0.000 plus or minus 0.000, 0.432 plus or minus 0.000.

* gemma 3: 4 b: 0.287 plus or minus 0.007, 0.256 plus or minus 0.004, 0.738 plus or minus 0.015, 0.097 plus or minus 0.005, 0.380 plus or minus 0.007.

* gemma 3: 12 b: 0.646 plus or minus 0.005, 0.444 plus or minus 0.004, 0.773 plus or minus 0.006, 0.592 plus or minus 0.007, 0.564 plus or minus 0.004 (highest F 1).

* qwen 3: 4 b: 0.479 plus or minus 0.014, 0.293 plus or minus 0.012, 0.537 plus or minus 0.027, 0.455 plus or minus 0.014, 0.379 plus or minus 0.017.

* qwen 3: 8 b: 0.669 plus or minus 0.006, 0.459 plus or minus 0.007, 0.641 plus or minus 0.013, 0.681 plus or minus 0.007, 0.535 plus or minus 0.008.

* deepseek-r 1: 1.5 b: 0.395 plus or minus 0.015, 0.080 plus or minus 0.013, 0.100 plus or minus 0.018, 0.519 plus or minus 0.021, 0.089 plus or minus 0.015.

* deepseek-r 1: 8 b: 0.459 plus or minus 0.023, 0.250 plus or minus 0.021, 0.413 plus or minus 0.034, 0.478 plus or minus 0.023, 0.311 plus or minus 0.026.

* deepseek-r 1: 14 b: 0.677 plus or minus 0.009 (highest Accuracy), 0.462 plus or minus 0.013 (highest Precision), 0.554 plus or minus 0.035, 0.729 plus or minus 0.011 (highest Specificity), 0.503 plus or minus 0.021.

* llama 3.2: 1 b: 0.015 plus or minus 0.003, 0.017 plus or minus 0.004, 0.041 plus or minus 0.009, 0.005 plus or minus 0.003, 0.024 plus or minus 0.005.

* llama 3.2: 3 b: 0.439 plus or minus 0.018, 0.330 plus or minus 0.012, 0.868 plus or minus 0.034, 0.259 plus or minus 0.018, 0.478 plus or minus 0.017.

* phi 3: 3.8 b: 0.233 plus or minus 0.019, 0.156 plus or minus 0.015, 0.362 plus or minus 0.041, 0.178 plus or minus 0.025, 0.218 plus or minus 0.022.

* phi 3: 14 b: 0.533 plus or minus 0.021, 0.300 plus or minus 0.021, 0.430 plus or minus 0.036, 0.577 plus or minus 0.028, 0.353 plus or minus 0.025.

Navigate back to Image 1:.

### Image 2: Long description

The multi-panel figure consists of four plots labeled a through d. Each plot shares a common y-axis listing twelve models from top to bottom: phi 3 14 b, phi 3 3.8 b, llama 3.2 3 b, llama 3.2 1 b, deepseek r 1 14 b, deepseek r 1 8 b, deepseek r 1 1.5 b, qwen 2 8 b, qwen 2 4 b, gemma 2 2 b, gemma 2 4 b, and gemma 2 1 b. The x-axis for all plots ranges from 0.0 to 1.0.

* Panel a Accuracy: Data points are clustered between 0.0 and 0.7. Deepseek r 1 14 b and qwen 2 8 b show the highest accuracy near 0.7. Llama 3.2 1 b is an outlier at 0.0.

* Panel b Sensitivity: Data points are more widely distributed. Gemma 2 1 b shows the highest sensitivity near 0.9, while llama 3.2 1 b remains near 0.0.

* Panel c Specificity: Deepseek r 1 14 b shows the highest specificity near 0.7. Llama 3.2 1 b and gemma 2 1 b are at 0.0.

* Panel d F 1 Score: Most models score between 0.1 and 0.6. Qwen 2 8 b and gemma 2 2 b show the highest performance in this metric near 0.55. Llama 3.2 1 b is at 0.0.

In all panels, blue dots represent the mean value, and horizontal error bars represent the 95 percent confidence intervals. Some models, like llama 3.2 1 b, consistently show zero or near-zero performance across multiple metrics.

Navigate back to Image 2:.

### Image 3: Long description

A square heatmap matrix with twelve model names on both the vertical y-axis and horizontal x-axis. The models listed are gemma 3 1 b, gemma 3 4 b, gemma 3 12 b, qwen 3 4 b, qwen 3 8 b, deepseek r 1 1.5 b, deepseek r 1 8 b, deepseek r 1 14 b, llama 3.2 1 b, llama 3.2 3 b, phi 3 3.8 b, and phi 3 14 b.

A color scale bar on the right ranges from negative 0.2 in light blue to 1.0 in dark red, with 0.0 represented by light gray.

Key data patterns include:

* A solid dark red diagonal line from top-left to bottom-right indicates a perfect correlation of 1.0 for each model with itself.

* High positive correlation clusters (orange to red) are visible between gemma 3 12 b, qwen 3 4 b, and qwen 3 8 b.

* Another cluster of high correlation exists between the deepseek r 1 variants (8 b and 14 b).

* Negative correlations (light blue) are sparse but notable between llama 3.2 1 b and gemma 3 12 b, as well as between llama 3.2 1 b and qwen 3 8 b.

* The gemma 3 1 b and gemma 3 4 b models show relatively low correlation (light gray to pale orange) with most other models in the set.

Navigate back to Image 3:.

